# Identification of Insecticidal Constituents of the Essential Oil of *Acorus calamus* Rhizomes against *Liposcelis bostrychophila* Badonnel

**DOI:** 10.3390/molecules18055684

**Published:** 2013-05-15

**Authors:** Xin Chao Liu, Li Gang Zhou, Zhi Long Liu, Shu Shan Du

**Affiliations:** 1 Department of Entomology, China Agricultural University, Haidian District, Beijing 100193, China; E-Mail: xchliu@yeah.net; 2 Department of Plant Pathology, China Agricultural University, Haidian District, Beijing 100193, China; E-Mail: lgzhou@cau.edu.cn; 3 College of Resources Science and Technology, Beijing Normal University, Haidian District, Beijing 100875, China

**Keywords:** *Acorus calamus*, *Liposcelis bostrychophila*, fumigant, contact toxicity, essential oil composition

## Abstract

The aim of this research was to determine the chemical composition of the essential oil of *Acorus calamus* rhizomes, its insecticidal activity against the booklouse, (*Liposcelis bostrychophila*) and to isolate any insecticidal constituents from the essential oil. The essential oil of *A. calamus* rhizomes was obtained by hydrodistillation and analyzed by GC-FID and GC-MS. A total of 32 components of the essential oil of *A. calamus* rhizomes was identified and the principal compounds in the essential oil were determined to be α-asarone (50.09%), (*E*)-methylisoeugenol (14.01%), and methyleugenol (8.59%), followed by β-asarone (3.51%), α-cedrene (3.09%) and camphor (2.42%). Based on bioactivity-guided fractionation, the three active constituents were isolated from the essential oil and identified as methyleugenol, (*E*)-methylisoeugenol and α-asarone. The essential oil exhibited contact toxicity against *L. bostrychophila* with an LD_50_ value of 100.21 µg/cm^2^ while three constituent compounds, α-asarone, methyleugenol, and (*E*)-methylisoeugenol had LD_50_ values of 125.73 µg/cm^2^, 103.22 µg/cm^2^ and 55.32 µg/cm^2^, respectively. Methyleugenol and (*E*)-methylisoeugenol possessed fumigant toxicity against *L. bostrychophila* adults with LC_50_ values of 92.21 μg/L air and 143.43 μg/L air, respectively, while the crude essential oil showed an LC_50_ value of 392.13 μg/L air. The results indicate that the essential oil of *A. calamus* rhizomes and its constituent compounds have potential for development into natural fumigants/insecticides for control of the booklice.

## 1. Introduction

The booklouse, *Liposcelis bostrychophila* Badonnel (Psocoptera: Liposcelididae) has a worldwide distribution and has been reported infesting domestic premises, raw material stores, manufacturing factories, and also museums [1]. These are tiny (approximately 1 mm in length), wingless, soft-bodied, light brown insects. Psocids used to be considered as nuisance pests rather than a cause of losses to stored commodities [2]. They were regarded as secondary pests, often overlooked due to their small size and the existence of other more damaging primary pests in cereal grains (e.g., maize weevils *Sitophilus zeamais*, rice weevils *S. oryzae* and lesser grain borer, *Rhyzopertha dominica*) [2]. However, currently, psocids are perhaps the most important category of emerging pests in stored grains and related commodities due to their small size and resistance to chemicals [2,3]. *L. bostrychophila* exhibits partenogenensis behavior, because all the offspring are female, so the rate of population increase is exponential and infestation could arise from a small indigenous population when favorable conditions occur [2]. Infestations of stored product insects currently are controlled by fumigation or insecticidal treatment of commodities and surfaces [4]. However, many problems are associated with heavy usage of these chemicals, such as the development of resistance, toxic residues in food, workers’ safety, and high cost of procurement [4]. These problems have necessitated a search for alternative eco-friendly insect pest control methods [5]. The use of essential oils or their constituents with low mammalian toxicity can effectively prevent and/or suppress insect pest especially in storage [6]. Investigations in several countries confirm that some plant essential oils not only repel insects, but possess contact and fumigant toxicity against stored product pests as well as exhibiting feeding inhibition or harmful effects on the reproductive system of insects [7]. Essential oils from many plants including medicinal herbs, spices and fruits have been evaluated with success for insecticidal/ repellency activity against stored product insects/mites, in some cases, have been proven more effective than traditionally used organophosphorus pesticides [8,9,10,11,12,13,14,15,16,17,18].

Besides insecticidal and repellent activities, essential oils from different plant sources have exhibited several biological activities, including antibacterial and antifungal [19], larvicidal [20,21,22], acaricidal [23], and nematicidal [24,25,26]. As a consequence, this vast arsenal of bioactive compounds has attracted significant and crescent attention of researchers in recent years [7]. During our screening program for new agrochemicals from Chinese medicinal herbs and wild plants, the essential oil of *Acorus calamus* L. (Acoraceae) rhizomes was found to possess strong insecticidal toxicity against the booklouse (*L. bostrychophila*). 

*A. calamus* (Sweet flag) is a wetland perennial monocot plant, whose scented leaves and rhizomes have been traditionally used medicinally against different ailments like, fever, asthma, bronchitis, cough and mainly for digestive problems such as gas, bloating, colic, and poor digestive function, and also used as a sedative, nerve tonic, antimicrobial agent, and expectorant [27,28]. Chemical composition of the essential oils obtained from leaves and rhizomes of *A. calamus* grown in different countries has been the subject of some studies and a great variation in chemical composition of the essential oils were observed [29,30,31,32,33,34,35,36,37]. The essential oil of *A. calamus* has been demonstrated to possess insecticidal activity against many species of insects, e.g., the kelp fly, *Coelopa frigida* [38], termite, *Odontotermes obesus* [39], the larger grain borer, *Prostephanus truncates* [40], the tobacco armyworm, *Spodoptera litura* [41] and *Callosobruchus phaseoli* [42]. Moreover, the ethanol extract of *A. calamus* exhibited strong repellency and contact effect to maize weevil, *S. zeamais* [43] and a supercritical fluid (CO_2_) extract of *A. calamus* possessed strong contact toxicity against German cockroaches, *Blattella germanica* [44]. However, a literature survey has shown that there is no report on insecticidal of *A. calamus* essential oil against the booklouse, thus we decided to investigate the chemical constituents and insecticidal activity of the essential oil of *A.* *calamus* against *L. bostrychophila* for the first time and to isolate any active constituent compounds from the essential oil.

## 2. Results and Discussion

### 2.1. Essential Oil Chemical Composition

The yield of *A. calamus* essential oil was 1.31% (V/W) and the density of the essential oil was determined to be 0.92 g/mL. GC-MS analysis of the essential oil of *A. calamus* rhizomes led to the identification and quantification of a total of 32 major components, accounting for 97.52% of the total components present (Table 1). 

**Table 1:** Chemical constituents of the essential oil derived from *Acorus calamus* rhizomes.

	RI *	Compound	Composition, %
1	931	α-Pinene	0.17
2	957	Camphene	0.55
3	981	*β*-Pinene	0.27
4	1030	(D)-Limonene	0.56
5	1032	1,8-Cineol	0.16
6	1094	Linalool	1.07
7	1146	Camphor	2.42
8	1179	Terpinen-4-ol	1.21
9	1191	α-Terpineol	0.66
10	1198	Estragole	0.89
11	1287	Bornyl acetate	0.13
12	1374	Copaene	1.08
13	1400	α-Funebrene	0.23
14	1406	α-Gurjunene	0.69
15	1409	α-Cedrene	3.09
16	1416	α-Bulnesene	0.32
17	1403	Methyleugenol	8.59
18	1422	β-Cedrene	1.52
19	1420	β-Caryophyllene	0.21
20	1454	α-Caryophyllene	0.19
21	1500	(*E*)-Methylisoeugenol	14.01
22	1458	Germacrene D	2.12
23	1504	Cuparene	0.73
24	1537	α-Cadinene	1.35
25	1557	β-Calacorene	0.51
26	1561	Germacrene B	0.26
27	1631	β-Asarone	3.51
28	1654	α-Cadinol	0.27
29	1678	α-Asarone	50.09
30	1685	Acorenone	0.23
31	1686	α-Bisabolol	0.13
32	1775	Acorone	0.11
		Total	97.52
		Monoterpenoids	7.20
		Sesquiterpenoids	13.04
		Others	77.28

* RI, retention index as determined on a HP-5MS column using the homologous series of *n*-hydrocarbons.

**Figure 1:**
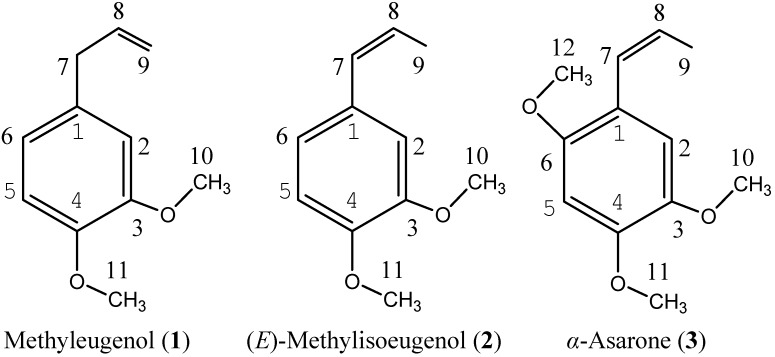
Main constituent compounds isolated from the essential oil of *A. calamus* rhizomes.

The principal compounds in the essential oil of *A. calamus* rhizomes were α-asarone (50.09%), (*E*)-methylisoeugenol (14.01%), and methyleugenol (8.59%) (Figure 1), followed by β-asarone (3.51%), α-cedrene (3.09%) and camphor (2.42%). 

Most of the essential oil components were phenylpropanoids (77.28%) and only 7.20% monoterpenoids and 13.04% sesquiterpenoids were identified (Table 1). The results are quite different from the previous reports. For example, preisocalamenediol (18.0%), acorenone (14.2%), shyobunone (13.3%) and cryptoacorone (7.5%) were major compounds of the essential oil of *A.* *calamus* collected from Quebec, Canada [34]. Preisocalamendiol (17.3%), isoshyobunone (13.0%), 1,4-(*trans*)-1,7-(*trans*)-acorenone (10.5%), camphor (5.9%) 2,6-diepishyobunone (2.6%) and β-gurjunene (2.5%) were the main components of the essential oil of *A. calamus* roots obtained from Turkey [31]. However, the essential oil of *A.* *calamus* rhizomes collected from Italy contained acorenone (21.6%), (*Z*)-sesquilavandulol (13.0%), shyobunone (7.0%), α-asarone (5.1%), and dehydroxyisocalamendiol (3.5%) [33] while the essential oil of *A.* *calamus* rhizomes collected from Lithuania contained acorenone (20.9%), isocalcamendiol (12.8%), shyobunone (7.8%), camphor (5.1%) and α-selinene (4.9%) [32]. Linalool (0.3%–12.0%), isoshyobunone (0.6%–9.4%), (*Z*)-methyl isoeugenol (2.4%–48.9%), (*E*)-methyl isoeugenol (1.1%–7.9%), β-asarone (20.5%–75.6%) and α-asarone (1.0%–16.0%) were reported to be the main components of *A.* *calamus* oil from Japan [45]. 

As for samples collected from China, β-asarone (85.68%) was demonstrated to be major constituent compound in one essential oil of *A. calamus* [30] and β-asarone (47.43%), calamenene (9.75%) and isocalamendiol (5.41%) were major compounds in the essential oil of *A.* *calamus* rhizomes collected from Hubei Province [35]. Li and Jiang [46] identified constituents of calamus oil isolated from different plant parts of *A. calamus* and found (*Z*)-methylisoeugenol (36.4% and 17.7%), acoragermacrone (4.1% and 7.4%) and δ-cadinene (4.1%–3.7%) as major components in leaf and rhizome oil, respectively. However, the essential oil of *A.* *calamus* rhizomes collected from Yunnan Province contained β-asarone (13.46%), α-asarone (7.22%), cedrol (6.19%), α-cedrene (5.61%), and berganotene (5.48%) while the sample from Sichuan Province had α-asarone (10.01%), β-asarone (9.16%), cedrol (7.94%), α-palchoulene (7.05%), and α-guaiadiene (6.68%) [36]. In a recent report, β-asarone (59.60%), 4-(5-hydroxy-2,6,6-trimethyl-1-cyclohexen-1-yl)-3-buten-2-one (19.49%), shyobunone (5.78%), dehydrofukinone (4.21%), and elemicin (3.26%) were the main constituent compounds in the oil harvested from Zhejiang Province [37]. Four types of *A. calamus* have been characterized: diploid (North America), triploid (Europe), tetraploid (East Asia, India and Japan) and hexaploid (Kashmir) [47]. Diploids are known to grow naturally in Eastern Asia (Mongolia and Central Siberian Plateau) and North America. Diploids do not produce β-asarone. The triploid cytotype probably originated in the Himalayan region, as a hybrid between the diploid and tetraploid cytotypes. Our samples probably belong to tetraploid or triploid because of containing β-asarone (3.51%). However, these differences of chemical composition of the essential oils might have been due to harvest time and local, climatic and seasonal factors as well as storage duration of medicinal herbs. For practical use, it is necessary to standardize the essential oil of the Chinese *A. calamus*.

### 2.2. Insecticidal Activities

The essential oil of *A. calamus* rhizomes exhibited contact toxicity against *L. bostrychophila*, with an LD_50_ value of 100.21 µg/cm^2^ (Table 2). When compared with the positive control pyrethrum extract (LD_50_ = 18.99 µg/cm^2^), the essential oil demonstrated five times less toxicity to *L. bostrychophila*. Three constituent compounds, α-asarone, methyleugenol and (*E*)-methylisoeugenol exhibited contact toxicity against the booklice with LD_50_ values of 125.73 µg/cm^2^, 103.22µg/cm^2^ and 55.32 µg/cm^2^, respectively (Table 2). 

**Table 2:** Contact toxicity and fumigant toxicity of the essential oil of *A. calamus* rhizomes and its constituents against *L. bostrychophila*.

	Treatment	LD_50_/LC_50_	95% FL *	Slope ± SE	Chi square (χ^2^)
Contact Toxicity (μg/cm^2^)	*A. calamus*	100.21	93.19–108.23	7.29 ± 0.89	23.52
	α-Asarone	125.73	114.45–136.89	11.03 ± 1.25	15.12
	Methyleugenol	103.22	94.87–112.19	9.47 ± 1.02	13.16
	(*E*)-Methylisoeugenol	55.32	50.89–59.63	5.34 ± 0.62	15.87
	Pyrethrum extract	18.99	17.56–20.06	7.64 ± 1.05	-
Fumigant (μg/L air)	*A. calamus*	392.13	365.76–430.54	6.81 ± 0.70	10.92
	α-Asarone	>2060.00	-	-	-
	Methyleugenol	92.21	83.29–100.78	3.50 ± 0.31	13.16
	(*E*)-Methylisoeugenol	143.43	131.19–157.65	4.13 ± 0.40	9.81
	Dichlorvos	1.35	1.25–1.47	6.87 ± 0.77	-

* Fiducial limits.

(*E*)-Methylisoeugenol possessed almost two times more toxicity than the two other constituents. It is suggested that (*E*)-methylisoeugenol is a major contributor to the insecticidal (contact) activity of the essential oil against the booklice, however, compared with pyrethrum extract (positive control), methylisoeugenol showed three times less toxicity. When compared with other essential oils, *A. calamus* essential oil possessed stronger contact toxicity than *Curcuma wenyujin* essential oil (with an LD_50_ value of 208.85 µg/cm^2^) [16] and showed the same level of contact toxicity as *Foeniculum vulgare* essential oil (LD_50_ = 90.36 µg/cm^2^) [17].

Methyleugenol and (*E*)-methylisoeugenol possessed fumigant toxicity against *L. bostrychophila* adults with LC_50_ values of 92.21 μg/L air and 143.43 μg/L air, respectively, while the crude essential oil of *A. calamus* rhizomes showed an LC_50_ value of 392.13 μg/L air (Table 2). However, α-asarone did not show fumigant toxicity at the tested concentrations. Compared with the positive control, dichlorvos (LC_50_ = 1.35 μg/L air), the two isolated constituent compounds exhibited almost 66 and 103 times less toxicity to *L. bostrychophila*, respectively. In previous reports, methyleugenol was demonstrated to possess insecticidal/acaricidal activity against several insects/mites, e.g., maize weevils (*S. zeamais*) and red flour beetles (*Tribolium castaneum*) [48], larval mosquitoes (*Culex pipiens pallens*, *Aedes aegypti*, *Ochlerotatus togoi*) [49], American cockroaches (*Periplaneta americana*) [50], mould mites (*Tyrophagus putrescentiae*) [51] and house dust mites (*Dermatophagoides farinae* and *D. pteronyssinus*) [52]. Methylisoeugenol also exhibited acaricidal activity against *D. farinae*, *D. pteronyssinus* and *T. putrescentiae* adults [53]. α-Asarone also demonstrated insecticidal activity against grain storage insects, *S. oryzae*, *Callosobruchus chinensis* and *Lasioderma serricorne* [54], larval mosquitoes (*C. pipiens pallens*, *A. aegypti*, *O. togoi*) [49] and agricultural insect pests such as *Nilaparvata lugens* and *Plutella xylostella* [55]. Asarones isolated from *A. calamus* rhizomes were potent growth inhibitors and antifeedants to the variegated cutworm, *Peridroma saucia* [56]. Moreover, Saxena *et al.* [57] found that asarone possessed insect chemosterilant properties, causing inhibition. The above findings suggest that that insecticidal activity especially fumigant activity of the essential oil of *A. calamus* rhizomes and its threeconstituent compounds against the booklouse is quite promising. As currently used fumigants are synthetic insecticides and the most effective fumigants (e.g., phosphine and MeBr) are also highly toxic to humans and other non-target organisms, the essential oil of *A. calamus* rhizomes and its three constituent compounds show potential to be developed as possible natural fumigants/insecticides for the control of *L. bostrychophila*. However, to develop a practical application for the essential oil and the isolated constituents as novel fumigants/insecticides, further research into the safety of the essential oil/compounds to humans is needed. Much work has been carried out to investigate the toxicity of α-and β-asarone. β-Asarone has been shown to cause cancerous tumours in the duodenal regions of rats [58]. However, medicinal plants containing these carcinogenic asarones are still widely used in traditional medicine in China, India and Mexico and also that in most European countries including Great Britain the presence of β-asarone as a flavouring agent is still permitted in alcoholic beverages up to a concentration of 1 mg/kg [58]. β-Asarone is only a minor compound (3.51%) found in our studied essential oil (Table 1). Additional studies on the development of formulations are also necessary to improve the efficacy and stability and to reduce cost.

## 3. Experimental

### 3.1. Plant Material and Essential Oil Extraction

Dried rhizomes (10 kg) of *A. calamus* were purchased from Anguo Chinese Medicinal Herbs Market (Anguo 071200, Baoding, Hebei Province, China). The rhizomes were ground to a powder using a grinding mill (Retsch Muhle, Germany). The species was identified by Dr. Liu, QR (College of Life Sciences, Beijing Normal University, Beijing, China), and the voucher specimens (CMH-ShuiChangPu-2012-09-003) were deposited in the museum of Department of Entomology, China Agricultural University. The powder was subjected to hydrodistillation using a modified Clevenger-type apparatus for 6 h and extracted with *n*-hexane. Anhydrous sodium sulphate was used to remove water after extraction. The essential oil was stored in airtight containers in a refrigerator at 4 °C for subsequent experiments.

### 3.2. Insects

The booklice (*L. bostrychophila*) were obtained from laboratory cultures in the dark in incubators at 28–30 °C and 70%–80% relative humidity and was reared on a 1:1:1 mixture, by mass, of milk powder, active yeast, and flour. All the containers housing insects and the Petri dishes used in experiments were made escape proof with a coating of polytetrafluoroethylene (Fluon®, Blades Biological Ltd., Cowden Edenbridge, Kent, UK). Laboratory bioassays were done within one week after adult collections.

### 3.3. Gas Chromatography-Mass Spectrometry

Components of the essential oil of *A. calamus* rhizomes were separated and identified by gas chromatography-flame ionization detection (GC-FID) and gas chromatography-mass spectrometry (GC-MS) using a Agilent 6890N gas chromatograph connected to an Agilent 5973N mass selective detector. The same column and analysis conditions were used for both GC-FID and GC-MS. They were equipped with capillary column with HP-5MS (30 m × 0.25 mm × 0.25 μm). The GC settings were as follows: the initial oven temperature was held at 60 °C for 1 min and ramped at 10 °C·min^−1^ to 180 °C where it was held for 1 min, and then ramped at 20 °C·min^−1^ to 280 °C and held there for 15 min. The injector temperature was maintained at 270 °C. The samples (1 μL, dilute to 1% with acetone) were injected, with a split ratio of 1:10. The carrier gas was helium at flow rate of 1.0 mL·min^−1^. Spectra were scanned from 20 to 550 *m/z* at 2 scans·s^−1^. Most constituents were identified by gas chromatography by comparison of their retention indices with those of the literature or with those of authentic compounds available in our laboratories. The retention indices were determined in relation to a homologous series of *n*-alkanes (C_8_–C_24_) under the same operating conditions. Further identification was made by comparison of their mass spectra with those stored in NIST 05 (Standard Reference Data, Gaithersburg, MD, USA) and Wiley 275 libraries (Wiley, New York, NY, USA) or with mass spectra from literature [59]. Relative percentages of the individual components of the essential oil were obtained by averaging the GC-FID peak area% reports. 

### 3.4. Purification and Characterization of Three Constituent Compounds

The crude essential oil of *A. calamus* rhizomes (25 mL) was chromatographed on a silica gel (Merck 9385, 1,000 g) column (85 mm i.d., 850 mm length) by gradient elution with a mixture of solvents (*n*-hexane, *n*-hexane-ethyl acetate). Fractions of 500 mL were collected and concentrated at 40 °C, and similar fractions according to TLC profiles were combined to yield 15 fractions. Fractions (5–8, 10, 12) that possessed contact toxicity, with similar TLC profiles, were pooled and further purified by preparative silica gel column chromatography (PTLC) with petroleum ether-acetone (50:1, v/v) until to obtain the pure compound for determining structure as methyleugenol (**1**, 0.7 g), (*E*)-Methylisoeugenol (**2**, 0.6 g) and α-asarone (**3**, 0.7 g). The structure of the compounds was elucidated based on nuclear magnetic resonance. ^1^H and ^13^C-NMR spectra were recorded on Bruker ACF300 [300 MHz (1H)] and AMX500 [500 MHz (1H)] instruments using CDCl_3_ as the solvent with TMS as internal standard. Electron impact mass spectra (EIMS) were determined on a Micromass VG7035 mass spectrometer at 70 eV (probe).

### 3.5. Isolated Constituent Compounds

*Methyleugenol* (**1**, Figure 1). A colorless oil, C_1__1_H_1__4_O_2_. ^1^H-NMR (500 MHz, CDCl_3_) δ (ppm): 6.81 (1H, d, *J* = 8.0, H-6), 6.74 (1H, dd, *J* = 8.0, 2.0, H-5), 6.71 (1H, d, *J* = 2.0 H-2), 5.96 (1H, ddt, *J* = 17.0, 10.4, 6.8, H-8), 5.07 (2H, m, H-9), 3.86 (3H, s, -OCH_3_), 3.87 (3H, s, -OCH_3_), 3.34 (2H, ddd, *J* = 6.8, 1.7, 1.4, H-7). ^13^C-NMR (125 MHz, CDCl_3_) δ (ppm): 148.8 (C-4), 147.3 (C-3), 137.7 (C-8), 132.6 (C-6), 120.4 (C-1), 115.6 (C-9), 111.8 (C-2), 111.2 (C-5), 55.9(-OCH_3_), 55.7 (-OCH_3_), 39.8 (C-7). EI-MS *m/z* (%): 178 (100), 163 (33), 151 (15), 147 (32), 135 (18), 131 (11), 107 (28), 103 (39), 91 (50), 77 (26). The spectral data matched with a previous report [59]. 

*(E)-Methylisoeugenol* (**2**, Figure 1). A colorless oil, C_1__1_H_1__4_O_2_. ^1^H-NMR (500 MHz, CDCl_3_) δ (ppm): 6.83 (1H, d, *J* = 8.0, H-6), 6.81 (1H, dd, *J* = 8.0, 2.0, H-5), 6.76 (1H, d, *J* = 2.0 H-2), 6.35 (1H, d, *J* = 11.2 Hz, H-7), 5.67 (1H, m, H-8), 3.81 (3H, s, -OCH_3_), 3.80 (3H, s, -OCH_3_), 1.87 (3H, d, *J* = 4.0 Hz, H-9). ^13^C-NMR (125 MHz, CDCl_3_) δ (ppm): 148.5 (C-4), 147.7(C-3), 130.7 (C-1), 129.5 (C-7), 125.4 (C-6), 121.4 (C-5), 112.2 (C-2), 110.1 (C-8), 55.9 (-OCH_3_), 55.8 (-OCH_3_), 14.36(C-9). EI-MS *m/z* (%): 178 (100), 163 (44), 147 (11), 135 (18), 131 (11), 115 (138), 107 (39), 103 (28), 91 (39), 79 (17), 77 (15).The spectral data matched with a previous report [60]. 

*α-Asarone* (**3**, Figure 1). A crystalline solid, C_12_H_16_O_3_. ^1^H-NMR (500 MHz, CDCl_3_) δ (ppm): δ: 6.95 (1H, s, H-2), 6.66 (1H, dd, *J* = 16, 1.6 Hz, H-7), 6.48 (1H, s, H-5), 6.09 (1H, dq, *J* = 16, 7.2 Hz, H-8), 3.90 (3H, s, 10-OCH_3_), 3.84 (3H, s, 11-OCH_3_), 3.81 (3H, s, 12-OCH_3_), 1.89 (3H, dd, *J* = 16, 7.2 Hz, H-10). ^13^C-NMR (125 MHz, CDCl_3_) δ (ppm): 159.9 (C-4), 148.9 (C-6), 143.5 (C-1), 125.2 (C-7), 124.5 (C-8), 119.2 (C-3), 109.9 (C-2), 98.1 (C-5), 56.8 (C-10), 56.6 (C-12), 56.2 (C-11), 18.9 (C-9). EI-MS *m/z* (%): 208 (100), 165 (38), 137 (21), 105 (13), 91 (19), 69 (25), 41 (11). The spectral data matched with a previous report [61]. 

### 3.6. Contact Toxicity with Treated Filter Paper

Range-finding studies were run to determine the appropriate testing concentrations of the essential oil of *A. calamus* rhizomes and pure compounds. The essential oil and compound were diluted in acetone. The filter paper with 3.5 cm in diameter (Whatman) was treated with 150 μL of the solution. Then the filter paper after treated with solid glue (Glue Stick, Jong Ie Nara Co., Ltd., Hong Kong) was placed in a Petri dish (3.5 cm in diameter) and 10 booklice were put on the filter paper by using a hair brush. The plastic cover with holes was put and all the Petri dishes were kept in incubators at 27–29 °C, 70%–80% r.h. for 24 h. Acetone was used as controls and pyrethrum extract was used as a positive control. Six concentrations (1.0%, 1.2%, 1.5%, 1.8%, 2.2%, and 4.5%) and five replicates of each concentration were used in all treatments and controls. Mortality of insects was observed and the observed data were corrected for control mortality using Abbott’s formula. The results from all replicates were subjected to probit analysis using the PriProbit Program V1.6.3 to determine LC_50_ values [62]. Pyrethrum extract (25% pyrethrin I and pyrethrin II) was purchased from Fluka Chemie (Buchs, Switzerland).

### 3.7. Fumigant Toxicity

Range-finding studies were run to determine the appropriate testing concentrations of the pure compounds and *A. calamus* essential oil. A filter paper strip (3.5 cm × 1.5 cm) treated with 10 μL of an appropriate concentration of test essential oil/compound in acetone. The impregnated filter paper was then placed in the bottom cover of glass bottle of 250 mL. The insects, 10 adults in a small glass bottle (8 mL), were exposed for 24 h and each concentration with five replicates. Six concentrations (0.7%, 1.0%, 1.5%, 2.2%, 3.3%, 5.0%) were used in all treatments and controls. Acetone was used as controls and dichlorvos was used as a positive control. The observed mortality data were corrected for control mortality using Abbott’s formula. The LC_50_ values were calculated by using Probit analysis [62]. Positive control, dichlorvos (99.9%) was purchased from Aladdin-Reagent Company (Shanghai, China).

## 4. Conclusions

The study indicates that the essential oil of *A. calamus* rhizomes and its constituent compounds, methyleugenol, (*E*)-methylisoeugenol and α-asarone have potential for development into natural fumigants/insecticides for control of the booklice.
